# CD51 labels periosteal injury-responsive osteoprogenitors

**DOI:** 10.3389/fphys.2023.1231352

**Published:** 2023-09-04

**Authors:** Ye Cao, Ivo Kalajzic, Brya G. Matthews

**Affiliations:** ^1^ Department of Molecular Medicine and Pathology, University of Auckland, Auckland, New Zealand; ^2^ Center for Regenerative Medicine and Skeletal Development, School of Dental Medicine, UConn Health, Farmington, CT, United States

**Keywords:** periosteum, fracture, Notch, SCA1, CD51, CD34

## Abstract

The periosteum is a critical source of skeletal stem and progenitor cells (SSPCs) that form callus tissue in response to injury. There is yet to be a consensus on how to identify SSPCs in the adult periosteum. The aim of this study was to understand how potential murine periosteal SSPC populations behave *in vivo* and in response to injury. We evaluated the *in vivo* differentiation potential of Sca1^−^CD51^+^ and Sca1^+^CD51^+^ cells following transplantation. *In vitro*, the Sca1^+^CD51^+^ population appears to be more primitive multipotent cells, but after transplantation, Sca1^−^CD51^+^ cells showed superior engraftment, expansion, and differentiation into chondrocytes and osteoblasts. Despite representing a clear population with flow cytometry, we identified very few Sca1^+^CD51^+^ cells histologically. Using a periosteal scratch injury model, we successfully mimicked the endochondral-like healing process seen in unstable fractures, including the expansion and osteochondral differentiation of αSMA^+^ cells following injury. CD51^+^ cells were present in the cambium layer of resting periosteum and expanded following injury. Sca1^+^CD51^−^ cells were mainly localized in the outer periosteal layer. We found that injury increased colony-forming unit fibroblast (CFU-F) formation in the periosteum and led to rapid expansion of CD90^+^ cells. Several other populations, including Sca1^−^CD51^+^ and CD34^+^ cells, were expanded by day 7. Mice with enhanced fracture healing due to elevated Notch signaling mediated by NICD1 overexpression showed significant expansion of CD51^+^ and CD34^hi^ cells in the early stages of healing, suggesting these populations contribute to more rapid healing. In conclusion, we demonstrate that periosteal injury leads to the expansion of various SSPC populations, but further studies are required to confirm their lineage hierarchy in the adult skeletal system. Our data indicate that CD51^+^ skeletal progenitor cells are injury-responsive and show good engraftment and differentiation potential upon transplantation.

## 1 Introduction

The periosteum is a critical source of skeletal stem and progenitor cells (SSPCs) that form callus tissue in response to injury. Fracture healing is delayed when periosteum is seriously damaged or removed, and periosteum retention can allow regeneration of areas of bone that would otherwise fail to regenerate (Zhang et al., 2005; Tate et al., 2007). Periosteal SSPCs usually remain quiescent during adulthood, but these cells can become active and proliferate extensively following fracture. Following this initial expansion phase, callus forms via a combination of endochondral-like callus formation and direct bone formation occurs beginning towards the end of the first week following injury in mice. This callus later becomes completely mineralized and is ultimately remodeled. Several key signaling pathways regulate periosteal response following healing, including Notch signaling (Dishowitz et al., 2012; Matthews et al., 2014; Novak et al., 2020). Overexpressing Notch 1 intracellular domain (NICD1) in αSMA^+^ cells improves the progression of fracture healing and mineralization *in vivo* when induced around the time of fracture (Novak et al., 2020). Both genetic and pharmacological inhibition of Notch signaling lead to impairments in fracture healing (Dishowitz et al., 2013; Wang et al., 2016; Novak et al., 2020). These results indicate that activation of Notch signaling promotes bone healing.

While it is well-accepted that the periosteum houses tissue-resident SSPCs, the identity of the population or populations that contribute to healing is still controversial. Numerous lineage-tracing reporters and cell surface markers have been proposed to prospectively identify SSPCs (Cao et al., 2020). Periosteal cells expressing periostin, cathepsin K, and paired-related homeobox 1 (Prx1) contribute to bone and cartilage during fracture healing (Kawanami et al., 2009; Wilk et al., 2017; Debnath et al., 2018; Duchamp de Lageneste et al., 2018; Julien et al., 2022; Chai et al., 2023). αSMA-CreER labels long-term, self-renewing osteochondral progenitors within the adult periosteum (Grcevic et al., 2012; Matthews et al., 2014; Matthews et al., 2021). It is also enriched in Mx1+ periosteal progenitor cells (Ortinau et al., 2019). The majority of injury-responsive periosteal progenitor cells are αSMA+, these cells rapidly expand and contribute to the majority of bone and a reasonable amount of cartilage formation (Grcevic et al., 2012; Matthews et al., 2014; Matthews et al., 2021).

Cell surface markers are useful for identifying SSPCs as they can be combined to refine populations and can be applied to many systems without the need for transgenic animals. Adult periosteum is enriched for many putative SSPC markers compared to bone marrow and endosteum in both humans and mice (Tournaire et al., 2020; Matthews et al., 2021; Cao et al., 2022). Similar marker combinations have been proposed for growth plate resident skeletal stem cells and periosteal stem cells, with presence of CD51, absence of CD90 and 6C3, and variable expression of CD105 and CD200, and these populations expand in response to fracture, particularly about a week after injury (Chan et al., 2015; Marecic et al., 2015; Debnath et al., 2018). We previously separated periosteal populations on the basis of Sca1 and CD51 expression. *In vitro*, Sca1^+^CD51^+^ and Sca1^−^CD51^+^ cells were both enriched for colony forming unit fibroblasts (CFU-F), but Sca1^+^CD51^+^ cells are multipotent progenitors, and Sca1^−^CD51^+^ cells are more restricted to osteoblast lineage differentiation (Matthews et al., 2021).

The aim of this study was to understand how potential periosteal SSPC populations behave *in vivo* and in response to injury. In particular we focus on Sca1^−^CD51^+^ and Sca1^+^CD51^+^ cells following transplantation. Using a periosteal scratch injury model, we successfully mimicked the fracture healing process, and investigated the response of these and other populations to injury. We also defined a population of progenitors that is the basis for enhanced healing due to NICD1 overexpression.

## 2 Materials and methods

### 2.1 Mice

All animals were obtained from either Vernon Jansen Unit at the University of Auckland or from UConn Health. All the handling and surgical procedures involving animals were approved by the University of Auckland Animal Ethics Committee (approval numbers 001940 and 002735), and UConn Health Institutional Animal Care and Use Committee (animal protocol number AP-200271-1023). Mice were housed in a controlled environment (12-h light/dark cycle, 22°C ± 2°C, and 55% ± 5% humidity) with *ad libitum* access to food and water.

The transgenic mice used in this study are listed in Table 1. αSMACreER/Tom/Col2.3GFP mice were generated using either Ai9 or A14 reporter animals (a gift from the University of Otago) (Madisen et al., 2010). To generate CAG-tdTomato mice with tdTomato (Tom) expression from the Rosa26 locus in all cells, Ai9 mice were bred with female HprtCre mice (Sinder et al., 2020). CAG-Tom mice were crossed with Col2.3GFP to generate CAG-Tom/Col2.3GFP donor cells for transplantation. αSMACreER mice were bred with Rosa^NICD1^ to generate αSMACreER/NICD1 (homozygous for NICD1) as described previously (Novak et al., 2020). All strains except NSG were maintained on a C57Bl/6J background. CreER was activated by administration of tamoxifen in corn oil (75 µg/g i.p.), the timing of tamoxifen for different studies is indicated in the figures or legends.

**TABLE 1:** Mouse lines used in this study.

Mouse line	Official name	Source/References
αSMACreER	B6.Cg-Tg(Acta2-cre/ERT2)1Ikal	Grcevic et al. (2012)
Col2.3GFP	B6.Cg-Tg(Col1a1*2.3-GFP)1Rowe/J	Kalajzic et al. (2002)
Ai9	B6.Cg-Gt(ROSA)26Sor^tm9(CAG-tdTomato)Hze^/J	Jax: 007909
Ai14	B6.Cg-Gt(ROSA)26Sor^tm14(CAG-tdTomato)Hze^/J	Jax: 007914
NSG	NOD.Cg-Prkdc^scid^ Il2rgt^m1Wjl^/SzJ	Jax: 005557
Rosa^NICD1^	Gt(ROSA)26Sor^tm1(Notch1)Dam/J^	Jax: 008159
HprtCre	129S1/Sv-Hprt^tm1(CAGcre)Mnn^/J	Jax: 004302

### 2.2 Periosteal cell isolation

Periosteum was isolated from the hind limbs and single cell suspensions generated similar to previous studies (Matthews et al., 2014). Briefly, tibias and femurs were roughly dissected, the epiphyses cut off, and bone marrow was flushed out with PBS. Remaining muscle was removed, then periosteum scraped, collected in a tube and enzymatically digested with either 0.2% collagenase P, 0.2% dispase II (Gibco, Life Technologies Corporation, Cat: 17105-041), 5% FBS or 0.05% collagenase P, 0.2% hyaluronidase (Sigma Aldrich, St Louis, MO, United States) in PBS for 1 hour at 37°C, 120 rpm. Tubes were mixed every 15 min to spread the tissues evenly in the digestion solution. The cell solution was then diluted in PBS, passed through cell strainer mesh, centrifuged, washed in 40 mL PBS, then resuspended.

### 2.3 Flow cytometry and cell sorting

Flow cytometry on periosteal cells was performed in a similar manner to our previous studies (Matthews et al., 2021). For detailed analysis of periosteal response to injury, we used a panel containing 15 cell surface markers in addition to GFP and tdTomato on the Cytek Northern Lights spectral cytometer, and a simpler panel on a BD LSRII. These and other reagents used are shown in Supplementary Tables S1–S3. TruStain blocking reagents were used in spectral cytometry analysis for 30 min at 4°C in the dark prior to the full stain. Antibody master mix was prepared for each panel by adding the brilliant violet antibodies to 5 μL Brilliant Stain Buffer (BD Biosciences, United States). The rest of the antibodies from the panel were then added to make up a final volume of 50 μL master mix. The cells were incubated for 30 min at 4°C in the dark with the master mix. DAPI (50 ng/mL final concentration) was added to each tube prior to analysis for dead cell exclusion.

Cell sorting was performed on a BD FACS Aria II using simplified stains. Cells were sorted with 100 µm or 130 µm nozzles into tubes containing αMEM 20% FBS.

### 2.4 Subcutaneous cell transplantation

Freshly sorted cells were combined with cultured bone marrow stromal cells (BMSCs) for subcutaneous transplant. Cells were sorted from samples generated from 2–3 animals. We sorted all available cells with the goal of obtaining 5,000 cells/population for transplant, and ultimately each implant contained 4,900–8,000 cells (Table 2). BMSCs from C57Bl/6 mice were cultured for 7 days prior to transplantation as previously described (Matthews et al., 2014). Following detachment with accutase, 750,000 BMSCs were added to the sorted populations, centrifuged, and resuspended prior to making gels for transplantation. Collagen gels (5 mg/mL, 100 µL volume) were made by mixing Rat Tail High Concentration Collagen I (Corning, United States, catalog 354249) with a suitable volume of 1M NaOH and cells in αMEM 10% FBS. Gels were incubated in a Petri dish at 37°C for at least 30 min prior to transplant. Transplantation was performed in isoflurane anesthetized NSG mice. The back of the mice was shaved and cleaned with 1% chlorhexidine, then a small incision created and pre-made collagen gels were placed in subcutaneous pockets on the flanks. Each recipient mouse received up to 2 implants in separate pockets. Mice were subcutaneously delivered up to 1 mg/kg body weight of buprenorphine twice a day over the first 2 days for post-operative analgesia.

**TABLE 2:** Donor cell (Tom^+^) counts pre and post transplantation.

	Cells/implant	Cells/ossicle
Sca1^−^CD51^+^	6,760 ± 759	11,504 ± 2,771
Sca1^+^CD51^+^	6,700 ± 670	3,677 ± 1,347

### 2.5 Periosteal scratch injury

The periosteal injury was performed under isoflurane anesthesia using a 25 G needle to poke through the skin and muscle and scratch the surface of tibia and femur. Both unilateral and bilateral injuries were performed. Buprenorphine analgesia was provided as described above.

### 2.6 Histology and immunostaining

Ossicles were dissected, fixed overnight in 4% paraformaldehyde, then washed with PBS prior to X-ray imaging. After X-ray, they were incubated in 30% sucrose overnight, and embedded in cryomatrix. 7 µm cryosections were collected at ∼30 µm intervals for the whole visible ossicle. Following DAPI staining, imaging was performed on a Zeiss Axioscan using the ×10 objective. Labeled cell counting was performed on all sections based on fluorescence colocalization with DAPI signal using ImageJ as described previously (Matthews et al., 2021). All sections from each ossicle were pooled, and the average donor cell numbers for each population were calculated. Labeled cell surface/bone surface measurements were performed on the central three sections of each ossicle. In order to measure the cell surface/bone surface, bone surface was drawn for each section along the inner (endosteal) and outer (periosteal) bone surfaces, Tom^+^ cell surface was drawn individually for each Tom^+^ cell, and added up as total Tom^+^ cell surface, GFP^+^ cell surface was drawn individually for each cell co-expressing Tom and GFP, and added up as total GFP^+^ cell surface. Tom^+^ cell surface/bone surface, and GFP^+^ cell surface/bone surface were calculated. At least three ossicles were analyzed for each transplanted population.

Mouse long bones were dissected and fixed in 4% PFA for 48–72 h, followed by sucrose overnight prior to embedding in cryomatrix, and sectioning with a tape transfer system as previously described (Dyment et al., 2016). For immunostaining, we utilized rat anti-Sca1 (ThermoFisher, catalog 14-5981, 1:100) and rabbit anti-CD51 (Abcam, catalog ab179475, 1:1,000) combined with the secondary antibodies donkey anti-rat Alexa-Fluor 647 (Jackson Immunoresearch, catalog J1712605153) and goat anti-rabbit Alexa Fluor 750 (ThermoFisher, catalog A-21039), all 1:500. Briefly, sections were permeabilized with 0.1% Triton X in PBS for 15 min, followed by 1 h incubation in Blocking Solution (5% BSA in 0.1% Tween 20/PBS (PBST)) with either 10% Normal Donkey Serum or Normal Goat Serum depending on which species the secondaries were raised in) at room temperature. After blocking, sections were incubated with primary antibody cocktail (made up in 1% BSA in PBST with either 2% Normal Donkey Serum or Normal Goat Serum) at 4°C overnight. Sections were incubated for 1 h in secondary antibody cocktail, then counterstained with 100 ng/mL DAPI for 5 min. Slides were washed three times in PBST for 5 min between each step. Following the last wash, slides were cover slipped with 50% glycerol in PBS.

After fluorescent imaging, histochemical staining was performed on the same section. Safranin O staining was performed as follows: sections were stained with Wiegert’s hematoxylin for 5 min, washed with tap water for 5 min, followed by distilled water for 1 min, stained with 0.2% Fast green for 15 min, washed with 1% acetic acid, then stained with 0.1% Safranin O for 1 min, washed with water for 5 min, and cover slipped with 50% glycerol in water.

### 2.7 *In vitro* assays

CFU-F assays were performed on freshly sorted cells. Cells were seeded at 20–50 cells/cm^2^ in αMEM 20% FBS and maintained in a humidified incubator at 37°C with 5% CO_2_ and 5% O_2_. Half media change was performed on day 4. Colonies were either fixed in 10% formalin and stained with crystal violet or underwent differentiation on day 7.

We induced differentiation of primary colonies using a combined osteogenic/adipogenic medium: αMEM 50 μg/mL ascorbate-2-phosphate, 5 mM β-glycerophosphate, 0.5 µM rosiglitazone, 1 µM insulin, and 10% FBS, and maintained at 37°C with 5% CO_2_. On differentiation day 2, plates were fixed in 10% formalin, stained for ALP, followed by Oil Red O, then crystal violet, as previously described (Matthews et al., 2014; Matthews et al., 2021).

### 2.8 Statistics

Data were analyzed using GraphPad Prism 9 (GraphPad Software, San Diego, CA) with *t*-test, one-way, or two-way analysis of variance (ANOVA) with appropriate *post hoc* tests. Exact n values are listed in figure legends. Values represent the number of biological replicates. In most flow cytometry experiments, 2-3 mice were pooled to generate a biological replicate. Flow and CFU-F data were generated with flow or sorts performed on the same day. Paired tests were used for flow cytometry datasets where different populations from one sample were evaluated. Each graph is presented as the mean ± standard error of the mean (SEM). *p* < 0.05 was considered as statistically significant.

## 3 Results

### 3.1 Sca1^−^CD51^+^ show superior expansion to Sca1^+^CD51^+^ cells upon *in vivo* transplantation

**FIGURE 1:**
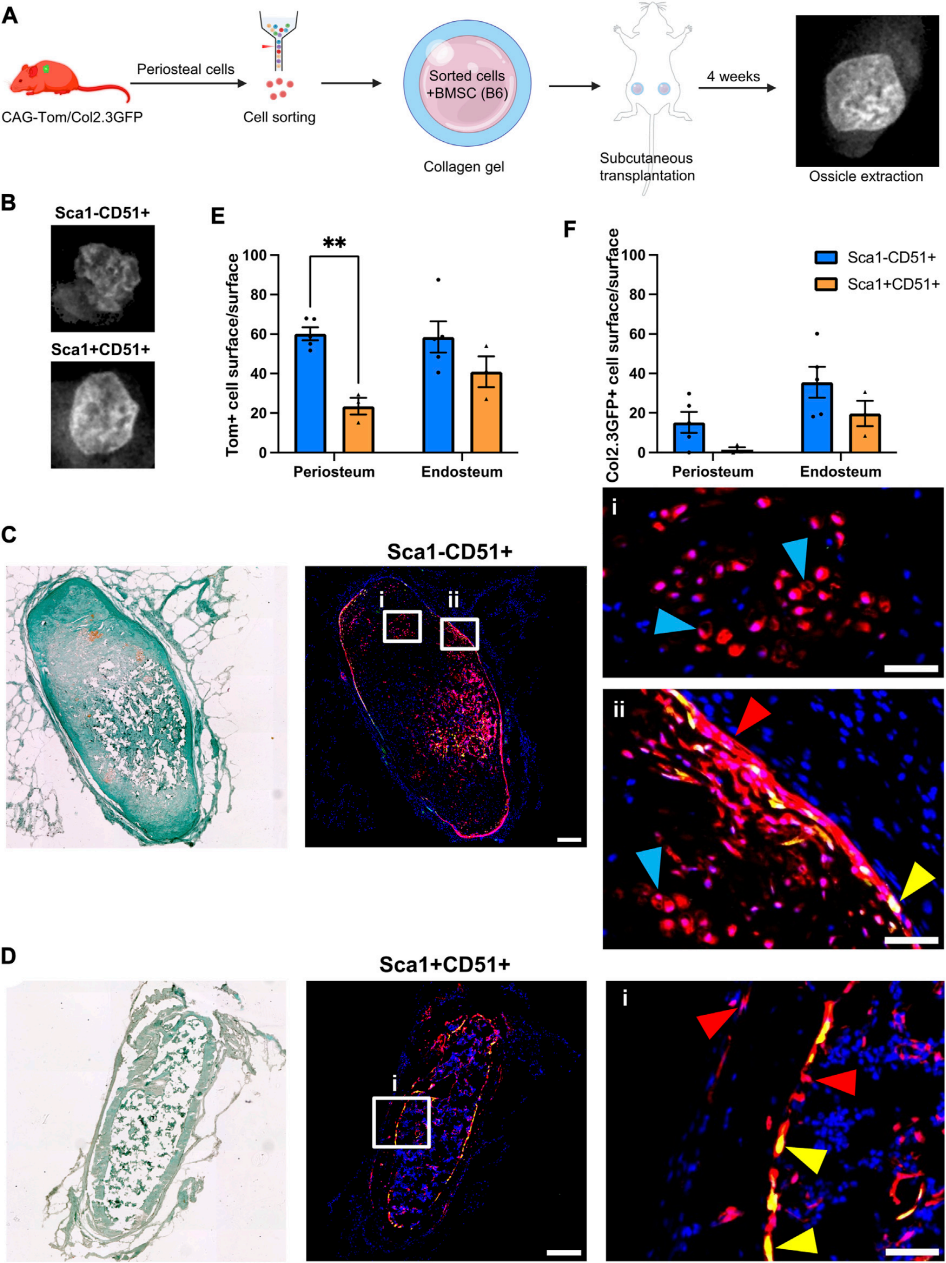
Periosteal Sca1^−^CD51^+^ cells contribute to osteoblasts and chondrocytes in ectopic bone **(A)** Experimental design. Periosteal donor cells were isolated from CAG-Tom/Col2.3GFP animals. Sorted populations (4,900–8,000 cells) were mixed with 750,000 bone marrow stromal cells (BMSCs) from wild type animals. After 4 weeks, implants were extracted. Representative image of BMSC only ossicle is shown. Figure partially created with BioRender. **(B)** X-rays of representative ossicles formed from the Sca1^−^CD51^+^, and Sca1^+^CD51^+^ cells. Representative sections showing cells derived from sorted **(C)** Sca1^−^CD51^+^ and **(D)** Sca1^+^CD51^+^ populations in ossicles. Magnified images indicating the red Tom^+^ donor cells (red arrowheads), and yellow donor cell-derived osteoblasts (yellow arrowheads) and chondrocytes (blue arrowheads) are shown in (i,ii). Sections were counterstained with DAPI. **(E)** tdTomato^+^ (donor), and **(F)** Col2.3GFP^+^ periosteum and (where present) endosteum surface was calculated (*n* = 4–5 implants/group). **p < 0.01 (*t*-test). Tom, tdTomato; DAPI, 4′,6-diamidino-2-phenylindole. Scale bars are 200 µm **(C,D)**, and 50 µm (i, ii).

We previously demonstrated that periosteal Sca1^+^CD51^+^ were enriched for cells capable of CFU-F formation and differentiation towards osteogenic and adipogenic lineages (Matthews et al., 2021). Sca1^−^CD51^+^ cells showed slightly lower CFU-F frequency and their differentiation was limited to the osteogenic lineage. We characterized the *in vivo* growth and differentiation capabilities of these populations following subcutaneous transplantation of CAG-Tom/Col2.3GFP donor cells (Figure 1A). We found ossicles from all the transplanted populations. The size and structure of the ossicles did not vary between the populations macroscopically (Figure 1B). All ossicles were comprised of an outer fibrous capsule/periosteal layer, bone, and central marrow compartments. Cells derived from the Sca1^−^CD51^+^ population survived and expanded well following transplantation, whereas the ossicles formed from the Sca1^+^CD51^+^ population contained fewer Tom^+^ donor cells within the sections we evaluated than we originally sorted for implantation (Table 2).

Tom^+^ cells were identified in all sections evaluated, with some expression of Col2.3GFP, indicating the ability of transplanted cells to engraft and undergo osteogenic differentiation (Figures 1C, D). Compared to Sca1^+^CD51^+^ donor cells, Sca1^−^CD51^+^ donor cells demonstrated higher engraftment (Figures 1E, F; Table 2), in contrast with our *in vitro* results (Matthews et al., 2021). Around 60% of the periosteum and endosteum surface was covered by cells derived from the Sca1^−^CD51^+^ population, and some of these cells differentiated into Col2.3GFP^+^ osteoblasts, covering 15% and 35% of the periosteum, and endosteum surface, respectively. Ossicles formed with Sca1^−^CD51^+^ cells contained bone, cartilage and a limited amount of bone marrow. Tom^+^ donor cells contributed to osteoblasts and chondrocytes, but it was unclear if they contributed to bone marrow stromal cells due to the limited amount of marrow present (Figure 1C). Despite lower engraftment, Sca1^+^CD51^+^ cells contributed to endosteal osteoblasts, endosteal and periosteal surface construction, and stromal cells in the bone marrow (Figure 1D). Ossicles formed with Sca1^+^CD51^+^ cells displayed a phenotype similar to ossicles formed from BMSCs only, containing bone marrow and endosteum partially covered by osteoblasts inside the cortical ring, and an outer fibrous capsule/periosteum. Approximately 20% and 40% of the periosteum and endosteum surface was covered by labeled cells, respectively (Figure 1E). Sca1^+^CD51^+^ derived Col2.3GFP^+^ osteoblasts covered around 15% of the endosteum surface, but they rarely formed osteoblasts on the periosteal surface (Figure 1F). These results indicate that Sca1^−^CD51^+^ cells show effective engraftment, expansion and differentiation towards osteogenic and chondrogenic lineages, while Sca1^+^CD51^+^ cells show poorer engraftment, in contrast to our previous *in vitro* studies, but are capable of osteogenic and stromal differentiation.

### 3.2 Evaluation of potential periosteal SSPC populations in mice following injury

**FIGURE 2:**
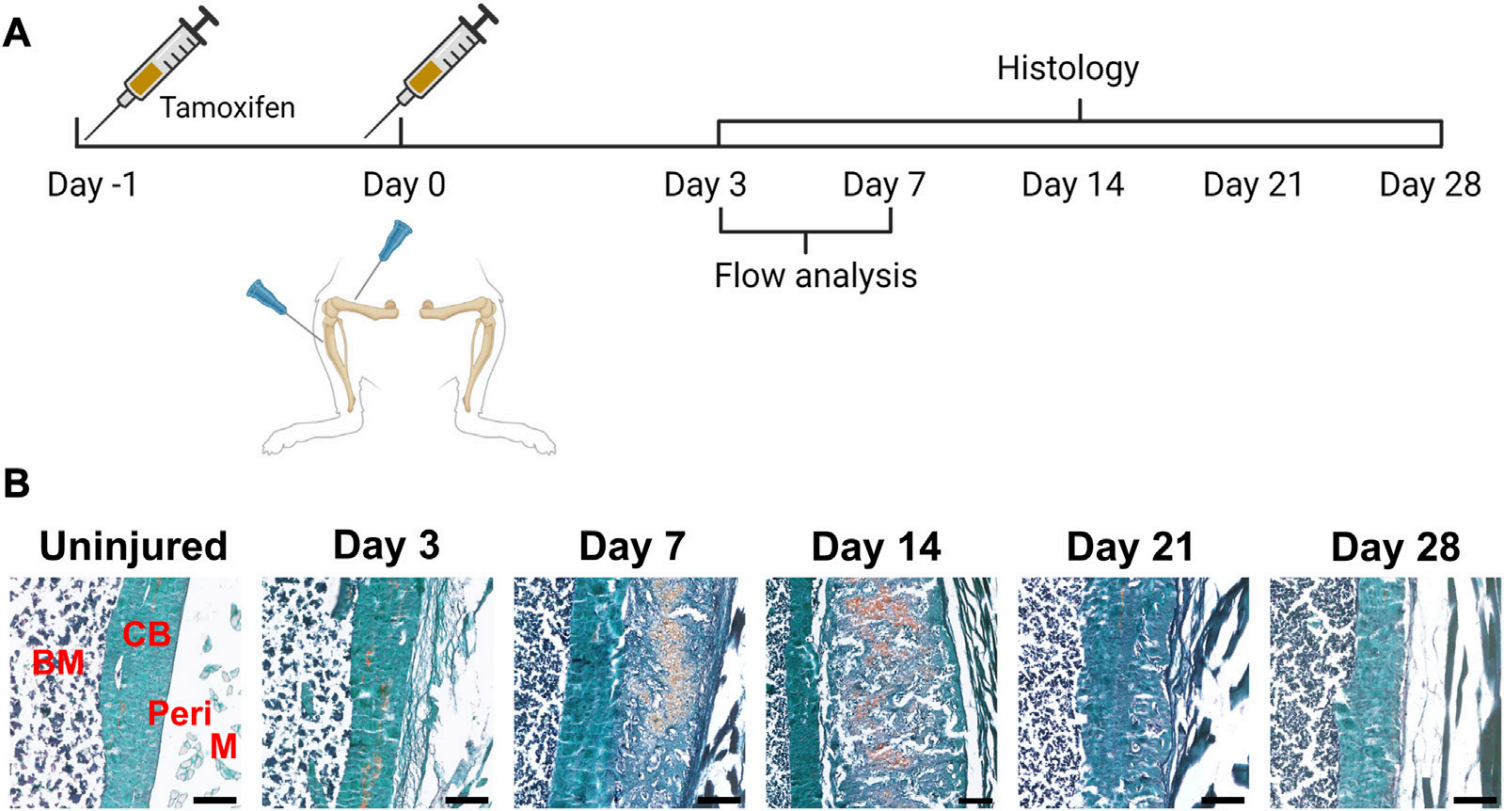
Time course of periosteal response to scratch injury. **(A)** Experimental design for histology and flow analysis of αSMACreER/Tom/Col2.3GFP mice following periosteal injury, created with BioRender. **(B)** Brightfield imaging of safranin O and fast green stained femur sections showing periosteal response following local injury at different time points (*n* = 3–5). BM, bone marrow; CB, cortical bone; Peri, periosteum (injured periosteum and healing response); M, muscle. Scale bars are 200 µm.

**FIGURE 3:**
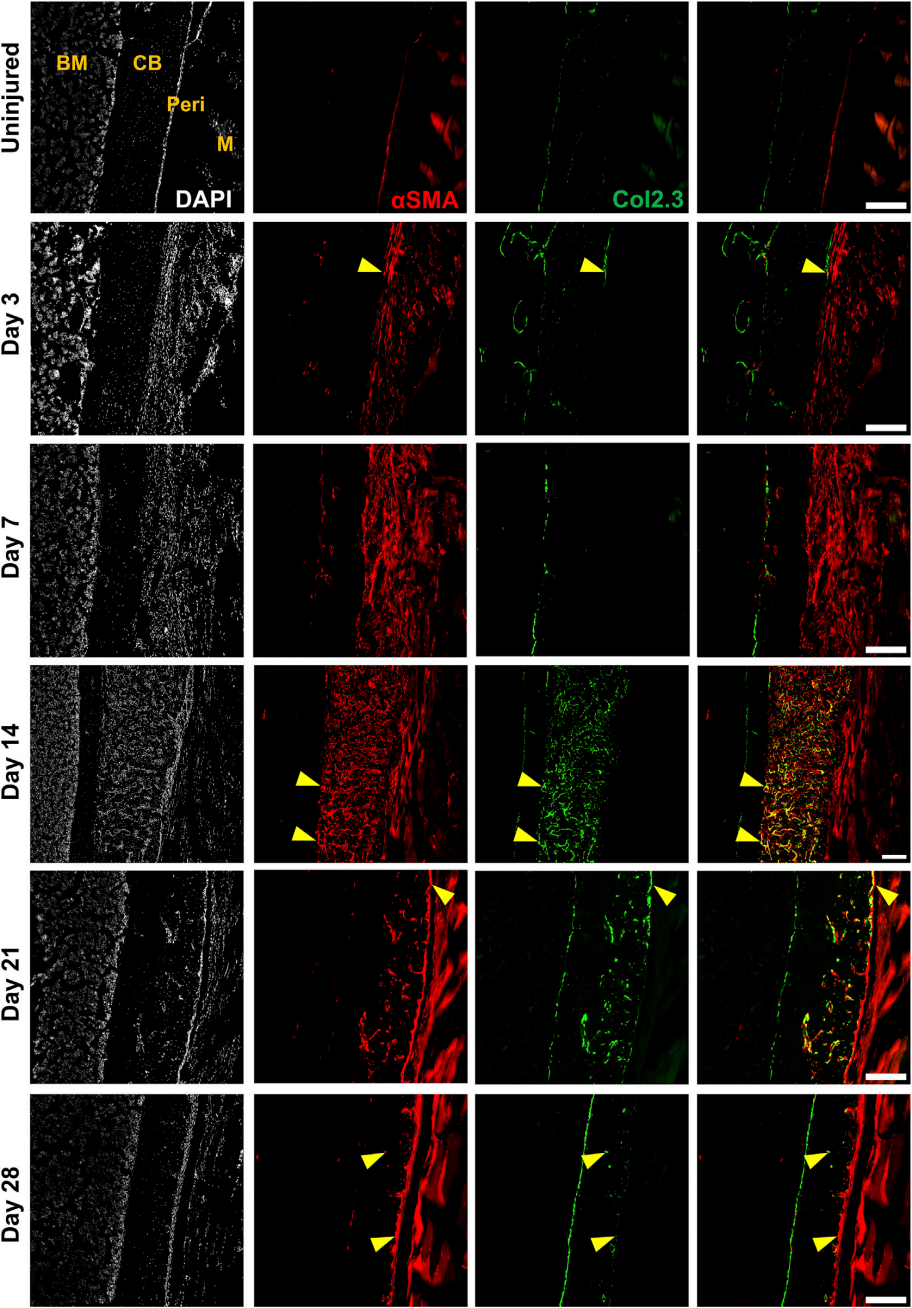
Alpha smooth muscle actin (αSMA) identifies injury-responsive periosteal stem and progenitor populations. Representative histology showing periosteum injury response compared to the uninjured femur at day 3 (*n* = 3), 7 (*n* = 4), 14 (*n* = 3), 21 (*n* = 4), and 28 (*n* = 2) following injury in αSMACreER/Tom/Col2.3GFP mice. DAPI (white), αSMA (red), Col2.3 (green) were labelled. αSMA cells rapidly expanded as soon as the injury occurred, these cells contributed to periosteum healing by giving rise to Col2.3GFP labelled osteoblasts (yellow arrowheads). BM, bone marrow; CB, cortical bone; Peri, periosteum (injured periosteum and healing response); M, muscle. Scale bars are 200 µm. DAPI, 4′,6-diamidino-2-phenylindole.

Next, we evaluated the response of these and other periosteal SSPC populations to injury. We evaluated the periosteum response following scratch injury at different time points with histology (*n* = 3–5 per group) (Figure 2A). This model enables damage to the periosteum without exposure of the bone marrow. We utilized adult αSMACreER/Tom/Col2.3GFP mice in order to localize the long-term periosteal progenitor cells labelled by αSMA, and osteoblasts labelled by Col2.3GFP. We successfully mimicked the endochondral healing process with the periosteal scratch injury (Figure 2B; Supplementary Figure S1). The periosteum layer was very thin without injury, but it was obviously thickened by day 3 following the scratch. Fibrocartilage formation was observed by day 7 between the bone surface and the thickened periosteum, meanwhile, new woven bone was found at the periphery of the injury, indicating the start of peripheral intramembranous bone formation. By day 14, bone formation gradually took over, with marrow infiltration filling the spaces between bone and cartilage tissues. Remodeling was underway by day 21, and by day 28, marrow infiltration almost disappeared, but there was still thickened periosteum and some areas of active remodeling at the injury site compared to uninjured bones. These results suggest that periosteum alone is healed through an endochondral process similar to semi-stabilized fracture (Matthews et al., 2014; Novak et al., 2020; Matthews et al., 2021). We also confirmed that cells labeled by αSMACreER dramatically expanded with periosteum injury by histology (Figure 3), consistent with our previous fracture studies (Matthews et al., 2014; Matthews et al., 2021). These cells were rapidly activated with periosteum expansion, contributed to new bone, cartilage and fibroblast formation, and were retained in the periosteum for at least a month after injury.

**FIGURE 4:**
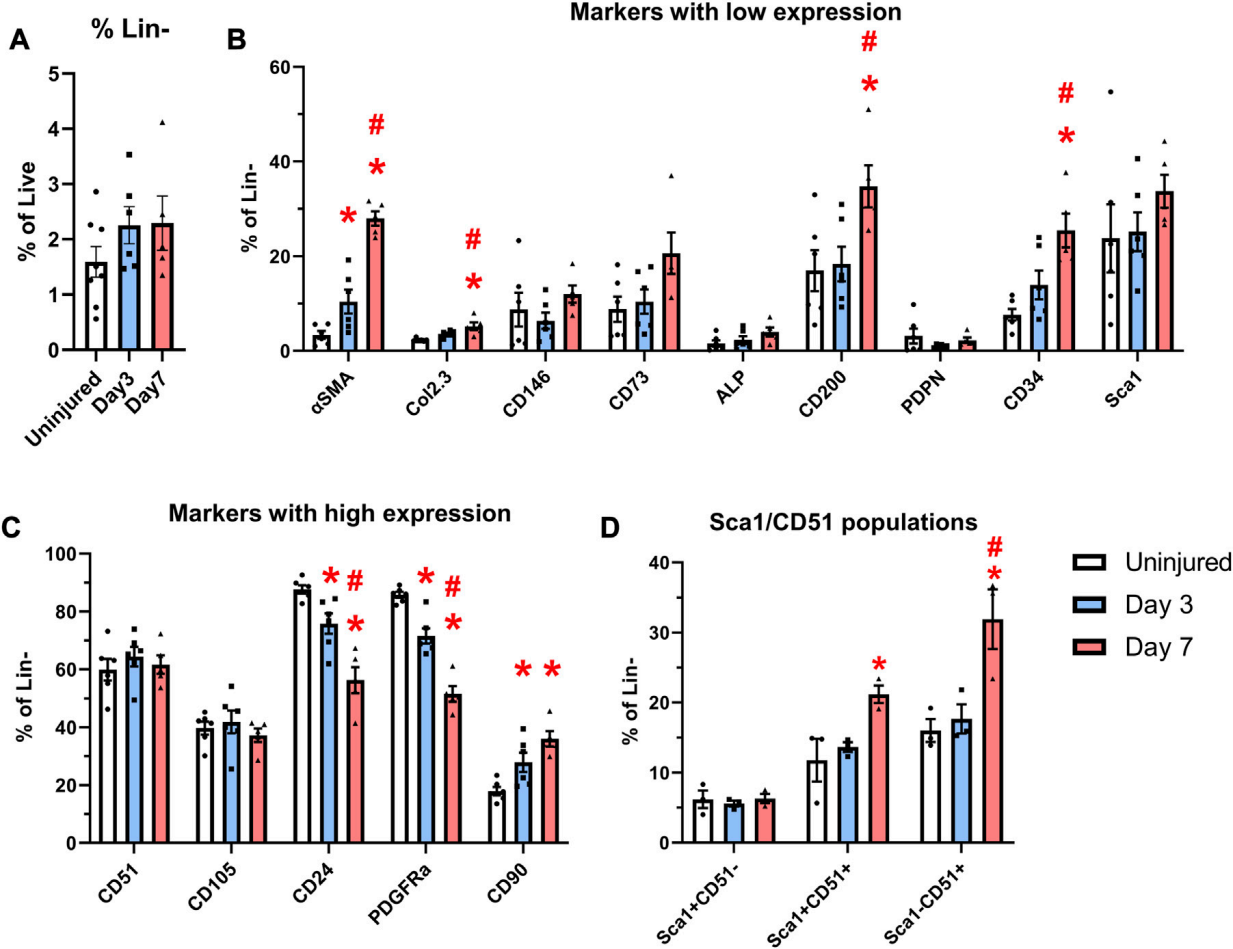
Expansion of cells expressing markers including CD90 and CD34 occurs after injury. αSMACreER/Tom/Col2.3GFP mice were treated with tamoxifen at day −1 and day 0, and had periosteal cells isolated 3 and 7 days later, uninjured αSMACreER/Tom/Col2.3GFP mice were treated with tamoxifen 1 and 2 days before harvesting. **(A)** The frequency of CD45/Ter119/CD31^−^ (Lin^−^) cells (*n* = 6–8). Expression of cell surface markers with low **(B)**, and high **(C)** expression in the periosteum following injury (*n* = 5-6). **(D)** Expression of populations expressing Sca1 and CD51 in a separate cohort of B6 mice (*n* = 3). *p < 0.05 compared to uninjured, #*p* < 0.05 compared to day 3 with one way ANOVA followed by Tukey’s *post hoc* test.

In order to characterize murine periosteal progenitor populations *in vivo* following injury, we performed spectral flow cytometry analysis at day 3 (inflammation stage) and day 7 (fibrocartilage stage). All events from each sample were recorded for analysis, and the average event numbers from different groups are shown in Table 3. Although the proportion of non-hematopoietic (Lin-) cells did not change with injury (Figure 4A), both live cell yields and Lin^−^ cell yields were enriched by day 3 and 7 compared to the uninjured group following injury (Table 3), indicating the expansion of the total periosteal cells, and Lin^−^ periosteal cells following injury consistent with the histology data.

**TABLE 3:** Periosteal Lin^−^ fraction event numbers isolated from different groups.

	Live cells	Lin- cells
Uninjured	207,347 ± 51,916	3,782 ± 1,197
Day 3	249,889 ± 25,504	5,621 ± 954
Day 7	353,219 ± 57,394	7,125 ± 564

Data shown as mean ± SEM.

We compared the expression of two transgenes and individual markers within the Lin^−^ populations at different time points following injury. Very few αSMA^+^ cells (around 3%) were present in the periosteum without injury (Figure 4B), which is consistent with our previous findings (Matthews et al., 2014; Matthews et al., 2021). After injury, the expression of αSMA significantly increased by day 3 and further at day 7. The proportion of Col2.3GFP^+^ osteoblasts was also increased after injury. The enrichment of αSMA^+^ cells and Col2.3GFP^+^ cells were also confirmed by histology (Figure 3). The frequency of CD24^+^ and PDGFRα^+^ cells significantly dropped by day 3 and was further decreased on day 7 (Figure 4C). CD90 expression was enriched at day 3 and day 7 compared to the uninjured group. Strong enrichment of CD200 and CD34 was also observed by day 7 post injury. In a different experiment using wild-type mice, the proportions of Sca1^+^CD51^+^ and Sca1^−^CD51^+^ cells were also increased at day 7 following injury (Figure 4D).

### 3.3 Localization of injury-responsive periosteal populations

**FIGURE 5:**
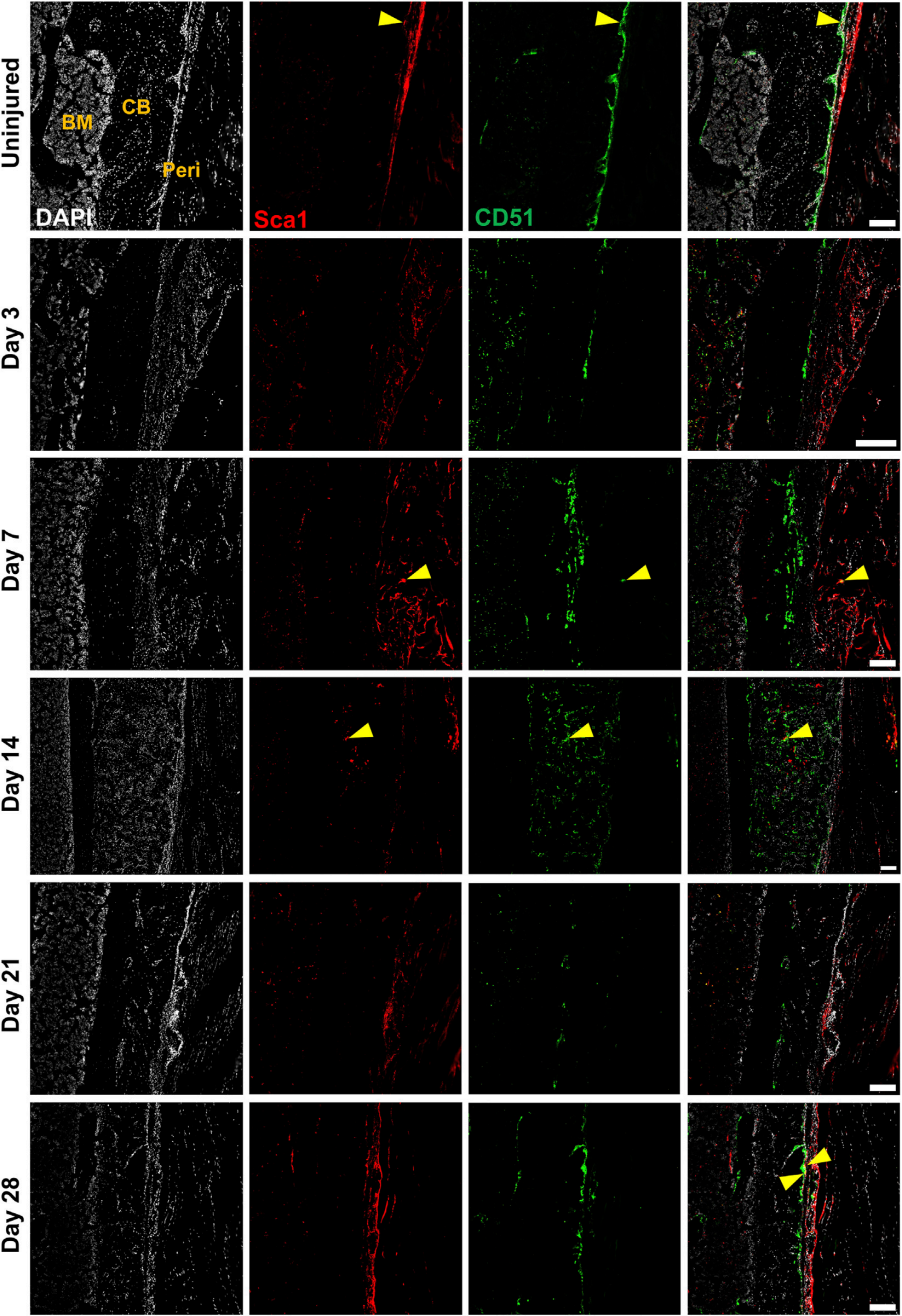
CD51^+^ periosteal cells expand in response to local injury. Representative histology showing periosteum injury response compared to the uninjured femur at day 3 (*n* = 3), 7 (*n* = 4), 14 (*n* = 3), 21 (*n* = 4), and 28 (*n* = 2) following injury. Sca1^+^ cells mainly resided in the outer layer of the periosteum and did not contribute much to healing; CD51 cells localized in the inner layer of the periosteum, contributed to bone and periosteum formation. Sca1^+^CD51^+^ cells (yellow arrowheads) were rare without injury and may decrease with injury. BM, bone marrow; CB, cortical bone; Peri, periosteum (injured periosteum and healing response). Scale bars are 200 µm. DAPI, 4′,6-diamidino-2-phenylindole.

**FIGURE 6:**
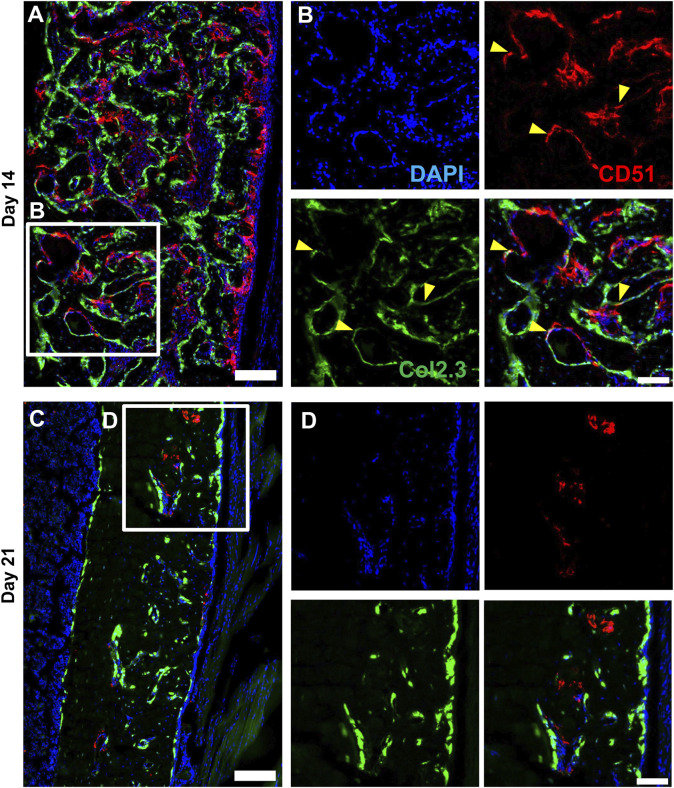
CD51 expression is detectable in some osteoblasts during active bone formation. Representative histology showing the localization of CD51 in relation to Col2.3GFP^+^ osteoblasts on day 14 **(A,B)** and day 21 **(C,D)** following injury (*n* = 3–4). DAPI (white), Col2.3 (green), CD51 (red) were labelled. CD51 labelled osteoblasts (yellow arrowheads) were found on day 14 post injury, but these cells disappeared on day 21. Scale bars are 200 µm **(A,C)** or 100 µm **(B,D)**. DAPI, 4′,6-diamidino-2-phenylindole.

We performed multicolor histology in αSMACreER/Tom/Col2.3GFP animals to localize selected injury-responsive periosteal progenitor populations. The contralateral uninjured femurs were used as the uninjured controls. Prior to injury, Sca1 was mainly expressed in the outer fibrous layer of the periosteum, next to muscle, whereas CD51 was mainly located in the inner cambium layer adjacent to bone (Figure 5; Supplementary Figure S2). By day 3 and day 7, in the periosteum, both Sca1 and CD51 cells were enriched with the expansion of periosteum (Figure 5). In contrast to flow analysis which consistently showed the presence of a Sca1^+^CD51^+^ population (Figure 4D), histologically, very few Sca1^+^CD51^+^ cells were present in the periosteum without injury, and these two markers were mostly expressed in separate layers after injury (Figure 5; Supplementary Figure S2). The highest CD51^+^ expression was found on post injury day 14, these cells were observed in the new bone, and inner periosteum area, but they were rarely present in the outer layer of the periosteum (Figures 5, 6). Some of these CD51^+^ cells also expressed Col2.3GFP (Figures 6A, B), but such co-expression disappeared a week later (Figures 6C, D), indicating that Sca1^−^CD51^+^ cells probably contribute to bone formation through forming osteoblasts, but they subsequently lose CD51 expression during maturation, suggesting that CD51 is present on osteoblast progenitors, and newly-formed osteoblasts, but not mature osteoblasts. This differs from our previous flow results showing 30%–60% of Col2.3GFP^+^ osteoblasts express CD51 (Matic et al., 2016; Matthews et al., 2021). Both CD51^+^ and Sca1^+^ cells were rare at post injury day 21, when periosteum shrunk, and the remodeling was mostly complete. By day 28, CD51^+^ cells returned to their original location in uninjured bones. CD51 may be expressed on the osteocytes, and in trans-cortical channels, but the majority of CD51^+^ cells resided in the inner layer of the periosteum. Unlike CD51, most Sca1^+^ cells resided in the outer periosteal layer during the whole healing process, except for at day 14 when some Sca1^+^ cells were present in the marrow infiltration area inside the new bone, indicating a potential stromal support function. CD51 and Sca1 were generally absent in fibrocartilage regions of the callus, and were never expressed on cells with chondrocyte morphology (Supplementary Figure S3). Our transplantation study showed that some Sca1^−^CD51^+^ cells were capable of chondrocyte formation, however, suggesting downregulation of CD51 during chondrocyte maturation. Overall, these results illustrate spatial separation of Sca1^+^ and CD51^+^ periosteal cells, with Sca1^+^ cells primarily present in the fibrous layer while CD51^+^ are cambium-resident and more closely-associated with tissue formation and remodeling.

### 3.4 Sca1^+^CD51^+^ and CD34^+^ cell expansion in a model of enhanced healing

**FIGURE 7:**
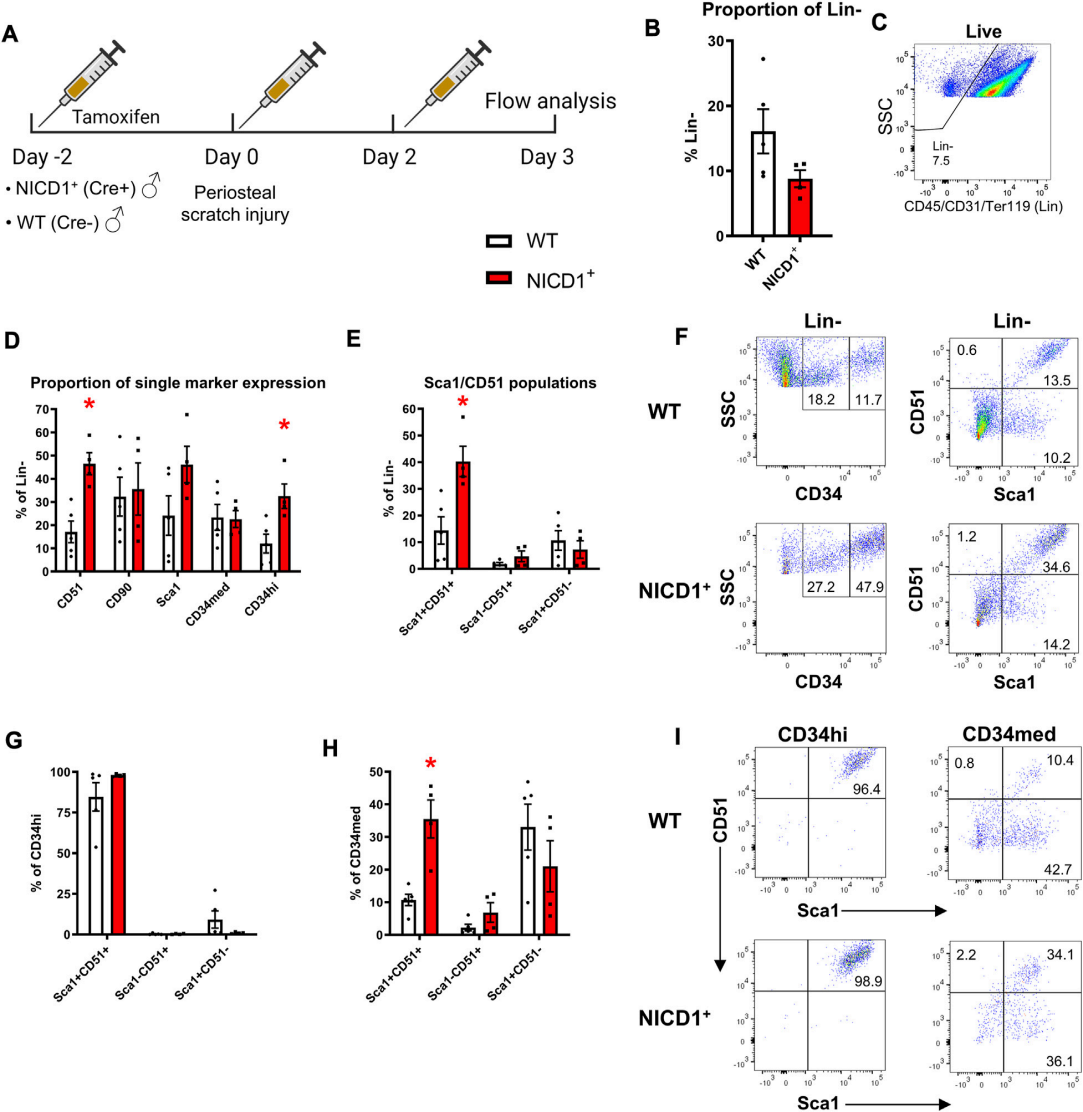
CD34^hi^ cells are stimulated with NICD1 overexpression following periosteal injury. **(A)** Experimental design of flow cytometry analysis on αSMACreER/NICD1 animals following periosteal injury (*n* = 4–5), partially created with BioRender. **(B)** The proportion of hematopoietic lineage negative (Lin^−^) from WT (Cre^−^) and NICD1^+^ (Cre^+^) mice, representative flow plot shown in **(C)**. **(D)** The proportion of single marker expression within the Lin^−^ fraction. **(E)** The proportion of Sca1/CD51 populations within Lin^−^. **(F)** Representative flow plots showing gating strategy of CD34^med^ and CD34^hi^ populations, and Sca1/CD51 populations within periosteal Lin^−^ of WT and NICD1^+^ mice. **(G)** The frequency of Sca1/CD51 populations within CD34^hi^ cells. **(H)** The frequency of Sca1/CD51 populations within CD34^med^ cells. **(I)** Representative flow plots showing Sca1/CD51 populations within CD34^hi^ and CD34^med^ populations of WT and NICD1^+^ mice. *p < 0.05 compared to WT with unpaired *t*-test. Percentages are specific to the sample. WT, wild type; NICD1, Notch intracellular domain 1.

Activation of Notch signaling stimulates fracture healing. In this experiment, we utilized αSMACreER/NICD1 animals that have an established enhanced fracture healing phenotype with the expansion of cells and osteoprogenitor during the early stage of fracture healing (Novak et al., 2020). All mice received three doses of tamoxifen to activate NICD1 expression in Cre^+^ animals and SSPC populations were evaluated at day 3 post injury (Figure 7A). Mice with overexpressed NICD1 did not show a significant change in Lin^−^ cells compared to wild type mice (Figures 7B, C). NICD1 overexpression led to expansion of CD34^hi^ and Sca1^+^CD51^+^ cells compared to wild-type (WT) Cre^−^ controls (Figures 7D–F). We found that CD34^med^ and CD34^hi^ cells had distinct cell surface marker phenotypes. CD34^hi^ cells were almost 100% Sca1^+^CD51^+^ (Figures 7G–I) while CD34^med^ contained all populations from the Sca1/CD51 combination.

**FIGURE 8:**
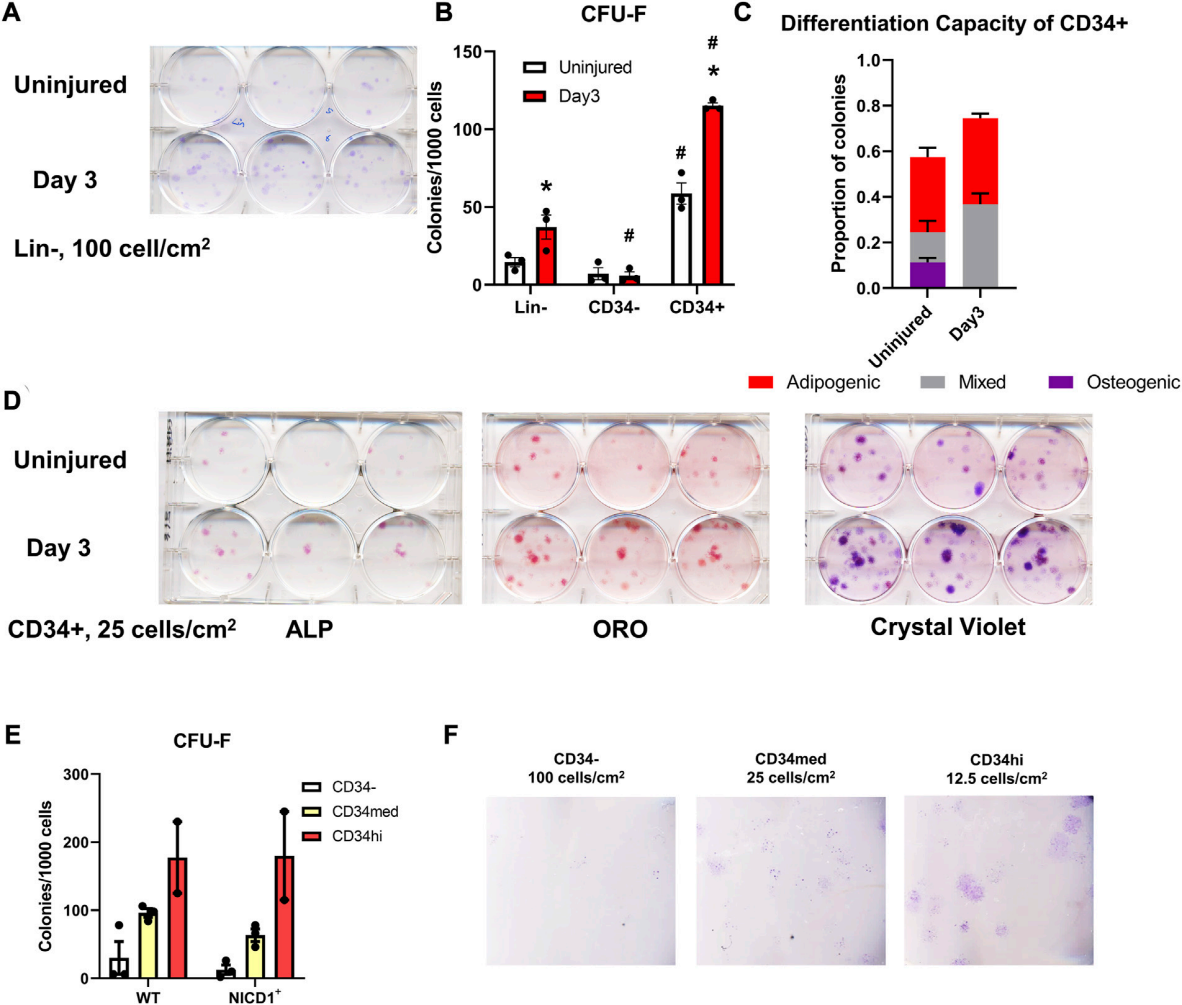
Injury enhances CFU-F formation overall and in CD34^+^ cells. Unilateral periosteal injury was performed on WT mice, and periosteal cells were harvested 3 days after injury, the uninjured cells were isolated from the matched uninjured legs (*n* = 3). **(A)** Representative plate image showing CFU-F of periosteal Lin-populations from uninjured and day 3 injury, stained with crystal violet, and **(B)** quantification of CFU-F for each population. CFU-Fs from the CD34^+^ cells were differentiated with dual-lineage media, and stained with alkaline phosphate (ALP, for osteogenesis), and oil red O (ORO, for adipogenesis), and the stained colonies were quantified **(C)**, representative stained colonies are shown in **(D)**. **(E,F)** Unilateral periosteal injury was performed on αSMACreER/NICD1 mice as indicated in Figure 7A, and periosteal cells sorted at day 3. **(E)** Quantification of CFU-F for CD34 populations in WT and NICD1+ animals (*n* = 2–3), and representative plate images with crystal violet staining of CFU-Fs are shown in **(F)**. Two-way ANOVA with Tukey’s *post hoc* test: *p < 0.05 compared to uninjured, #*p* < 0.05 compared to Lin^−^ within the same time point. Lin^−^: hematopoietic lineage negative.

In *ex vivo* assays, periosteal Lin^−^ cells isolated from WT mice showed greater CFU-F forming ability at day 3 post scratch injury than those from uninjured mice (Figures 8A, B). CD34^−^ cells contributed minimally to CFU-F formation *in vitro*, whereas the CD34^+^ population formed around 4× more CFU-F than the Lin^−^ cells without injury (Figure 8B). Three days post injury, the frequency of CFU-F formation in periosteal CD34^+^ cells was almost doubled compared to uninjured CD34^+^ cells, indicating better expansion and proliferation potential of skeletal progenitor cells during the early stages of the periosteum healing process. We also examined the differentiation capacity of the CD34^+^ cells with or without injury. CD34^+^ cells from injured periosteum formed adipocytes rapidly, therefore, in order to fairly compare their differentiation potential, cells were fixed and stained at day 2 post differentiation when massive adipocyte colonies were observed. We found that some periosteal CD34^+^ cells with and without injury were bi-potent, containing colonies with osteogenic, adipogenic, and combined potentials (Figures 8C, D). It is not surprising that some colonies were undifferentiated on day 2 after differentiation induction. These results suggest that the periosteal CD34^+^ cells are immature progenitors that can be stimulated with periosteum injury.

To better understand the functions of different CD34 populations, we isolated CD34^−^, CD34^med^, and CD34^hi^ populations from αSMACreER/NICD1 mice 3 days after periosteum injury and investigated their colony-forming potential (Figures 8E, F). CD34^hi^ cells exhibited the best CFU-F formation ability, but NICD1 overexpression did not alter the CFU-F formation. These results suggest that the expression of CD34 and CD51 is rapidly stimulated with injury when NICD1 is overexpressed around the time of injury. CD34^+^ cells exhibit better expansion, proliferation and differentiation potential following periosteal injury.

## 4 Discussion

In this study we sought to identify injury-responsive stem and progenitor populations in adult murine periosteum. Our studies indicate that CD51 is expressed on periosteal stem and progenitor cells. Many groups use CD51 as one of the markers for skeletal stem and progenitor phenotype (Chan et al., 2009; Pinho et al., 2013; Chan et al., 2015; Green et al., 2021). We have previously reported that all periosteal SSPCs capable of CFU-F formation expressed CD51, although CD51 also labelled a large portion of osteoblasts *in vivo* (Matthews et al., 2021). We separated CD51^+^ cells on the basis of Sca1 expression, which in our previous study enabled separation of Sca1^+^CD51^+^ CFU-F with multilineage differentiation potential from Sca1^−^CD51^+^ cells which formed fewer CFU-F and showed restricted potential to the osteoblast lineage (Matthews et al., 2021). Surprisingly, Sca1^−^CD51^+^ cells isolated from resting periosteum showed much greater engraftment and expansion *in vivo* than the Sca1^+^CD51^+^ cells that appear more stem-like *in vitro*. Notably, we previously observed good engraftment and contribution to new bone formation when mature osteoblasts or bone lining cells identified by *Dmp1* expression were transplanted (Matic et al., 2016). While this ectopic bone formation model does not directly replicate any clinical scenario, this data suggest that progenitor cells may be more effective than stem cells for transplantation in scenarios where rapid expansion and tissue formation are required to enable one-off skeletal regeneration.

Consistent with our previous flow data (Matthews et al., 2021), immunostaining results confirmed that Sca1 expression is enriched in the periosteum. However, these cells rarely co-expressed CD51, and resided primarily in the outer periosteum which thought to house fibroblasts rather than stem and progenitor cells. Single cell RNAseq analysis of periosteal cells suggested that Sca1 is a differentiated periosteal cell marker (Debnath et al., 2018). There appears to be differences in sensitivity between flow and immunostaining studies which make it difficult to localize the Sca1^+^CD51^+^ population *in vivo*. *In vivo*, CD51^+^ cells demonstrated cambium layer localization, which is more consistent with what we expected from periosteal stem and progenitor cells. Notably, we rarely found CD51^+^ osteoblasts *in vivo* except during the most active phase of bone formation in the callus suggesting that CD51 is downregulated as osteogenic differentiation progresses. Single cell RNAseq analysis of bone marrow cells suggested that CD51 is enriched in Col2.3Cre labelled cells compared to cells expressing LepRCre and VE-CadCre (Tikhonova et al., 2019). Among the three clusters deriving from the Col2.3Cre^−^ labelled cells, CD51 is downregulated in the cluster that appears most likely to represent true osteoblasts based on high expression of *Ibsp* and *Bglap*. This agrees with our data that CD51 expression diminishes with osteoprogenitor maturation. Another single cell RNAseq analysis of bone marrow stroma also suggested that CD51 expression is enriched in the osteogenic lineage cell cluster (cluster 7) (Baryawno et al., 2019). Both studies show fairly low expression of CD51 in around a third of cells within osteoblast clusters, consistent with our previous flow analysis of Col2.3GFP^+^ endosteal cells (Matic et al., 2016; Matthews et al., 2021).

We utilized a periosteal scratch injury as an alternative to creating a full fracture. Consistent with previous reports, this model recapitulated the healing process following generation of a semi-stabilized fracture, despite the absence of instability (Colnot, 2009; Hagiwara et al., 2015). The early phases of healing showed a remarkably similar time course to full fracture healing, although the final remodeling stage appeared to progress more quickly, presumably because repositioning and remodeling of the original cortical bone was not required. Consistent with our previous findings in fracture, αSMA labels periosteal progenitor cells that expand dramatically after injury and give rise to osteoblasts, chondrocytes, and osteocytes (Matthews et al., 2021). We reasoned that stem and progenitor cells that are critical for healing should begin to expand in the early stages of fracture prior to the initiation of fibrocartilage formation. The only marker apart from αSMA-derived cells that showed consistent expansion at day 3 was CD90. CD90 is considered a marker of osteochondrogenic progenitors in fetal and neonatal skeletal tissues, and more immature stem and progenitor are often reported to be CD90^−^ (Chan et al., 2009; Chan et al., 2015; Debnath et al., 2018). We have found that CD90 enriches for periosteal CFU-F in both mice and humans (Matthews et al., 2021; Cao et al., 2022). A study using single cell RNAseq on cells from resting periosteum showed co-expression of CD90 with Sca1 and CD34 in what their analysis identified as an undefined non-osteoblastic mature periosteal cell type (Debnath et al., 2018). We did not find any difference in CD90^+^ cell response in our model of enhanced healing.

Several cell populations, including Sca1^+^CD51^+^, Sca1^−^CD51^+^ and CD34^+^ cells showed enhanced expansion by day 7 after injury. This is consistent with other studies showing strong expansion of various proposed SSPC populations by about a week after fracture (Marecic et al., 2015; Debnath et al., 2018). We noted the appearance of a CD34^hi^ population primarily after injury. CD34 was traditionally considered a negative marker for SSPCs (Viswanathan et al., 2019), but more thorough analyses suggest that CD34 is present in at least some SSPC types *in vivo* including those in the periosteum, but is downregulated in culture (Ball et al., 2011; Abdallah et al., 2015; Cao et al., 2022). Julien et al. reported the skeletal stem/progenitor cluster is highly enriched for CD34 compared to the macrophages or osteoclasts cluster from single-cell RNAseq analysis (Julien et al., 2022). In this study, we found that the CD34^+^ population was relatively rare in intact adult periosteum, but its expression increased significantly by day 7 following local injury. Periosteal CD34^+^ cells expanded and differentiated faster 3 days after injury, these cells were osteogenic and adipogenic *in vitro*. CD34^hi^ cells in particular were much more common in our Notch-mediated model of enhanced healing. Future studies will be needed to address localization of the CD34^+^ populations, however strong expression of CD34 in other cell types including endothelial cells complicates this analysis.

Notch signaling controls bone growth and homeostasis in mice and humans. Dishowitz et al. (2013) inhibited Notch signaling systemically using Mx1Cre; dnMAML mice which led to impaired fracture healing with prolonged inflammation. A different model of impaired Notch signaling, Prx1Cre; RBPjk^fl/fl^, had fracture non-union (Wang et al., 2016). Pharmacological Notch1 inhibition also impaired fracture healing, albeit to a lesser extent (Novak et al., 2020). Conversely, overexpressing NICD1 in αSMA^+^ cells at the early stages of fracture accelerates the progression of fracture healing in male mice (Novak et al., 2020). Using similar injury-related activation of NICD1, we found that the frequency of cells expressing CD51 was enriched with NICD1 overexpression at day 3 following periosteal injury. Sca1^+^CD51^+^ and CD34^hi^ cells were also highly enriched with NICD1 overexpression. These results indicate that greater or earlier expansion of CD51^+^, Sca1^+^CD51^+^, and CD34^hi^ cells may improve bone healing. CD34^hi^ was a refined Sca1^+^CD51^+^ subpopulation that enriched for CFU-F formation.

This study has several limitations. We performed *in vitro* differentiation assays on osteogenesis and adipogenesis with selected populations, but not chondrogenesis in parallel due to insufficient cell density and large differences in differentiation conditions. It is surprising that periosteal cells undergo adipogenesis so readily given this is not seen *in vivo*, but is consistent with our recent studies of human periosteum (Cao et al., 2022). We performed a limited number of *in vivo* transplantation assays using subcutaneous transplantation with carrier cells. It is still unclear whether this assay, or others using different types of transplantation, accurately reflect the *in vivo* potential of cell populations. In addition, we only evaluated cell fate at a single time point so the Sca1^−^CD51^+^ derived ossicles can be either more immature, developing bone and marrow infiltration later than the other ossicles, or more osteogenic, never forming the same amount of stromal cells as the other populations. Our study clearly illustrates the challenges integrating data from *in vitro*, *in vivo* and *in situ* studies to understand the function and potential of stem and progenitor cell populations. It is still unclear how removing periosteal cells from their niche affects their behavior, presenting a limitation for any type of *ex vivo* or transplantation studies. The periosteum scratch injury avoids the direct infiltration of bone marrow cells as part of the callus, although these cells generally appear to make a very minor direct contribution (Colnot, 2009). Nonetheless, we cannot avoid some injury to the neighboring muscle during this process.

In conclusion, we have confirmed that local injury to the periosteum heals with a similar process to semi-stabilized fracture healing through endochondral ossification. Injury leads to expansion of various SSPC populations, and an overall increase in the frequency of CFU-Fs. Sca1^−^CD51^+^ cells are osteochondral progenitors resident specifically in the cambium layer of the periosteum that expand and contribute to bone and cartilage upon transplantation and likely do the same in the context of injury. Sca1^+^CD51^+^ cells could not be localized histologically, and despite high expansion and differentiation potential *in vitro* and following injury, they show poorer expansion or survival following transplantation making the utility of Sca1/CD51 combination in identifying periosteal SSPC populations uncertain without additional markers. Histologically Sca1 was primarily detectable in the outer fibrous layer of the periosteum that is not thought to house SSPCs. Further refinement and characterization of populations including Sca1^−^CD51^+^ and CD34^hi^ cells is important in order to confirm their lineage hierarchy in the adult skeletal system, and ensure that mature cells like osteoblasts are excluded. Finally, our data suggests that skeletal progenitor cells may be more effective than stem cells for regenerative uses that do not require long-term engraftment.

## Data Availability

The raw data supporting the conclusion of this article will be made available by the authors, without undue reservation.
