# Supplementary material for: CD51 labels periosteal injury-responsive osteoprogenitors
Source: Front Physiol. 2023 Sep 4;14:1231352. doi: 10.3389/fphys.2023.1231352 (PMC10507171; doi:10.3389/fphys.2023.1231352)
Supplement: Supplementary file 1 [file DataSheet1.DOCX]

Supplementary Material

CD51 labels periosteal injury-responsive osteoprogenitors

Ye Cao^1^, Ivo Kalajzic^2,*^, Brya G. Matthews^1,2,*^

*** Correspondence:**

*** Correspondence:**Ivo Kalajzic
ikalaj@uchc.edu

Brya G. Matthews
brya.matthews@auckland.ac.nz

# Supplementary Figures and Tables

## Supplementary Figures

**
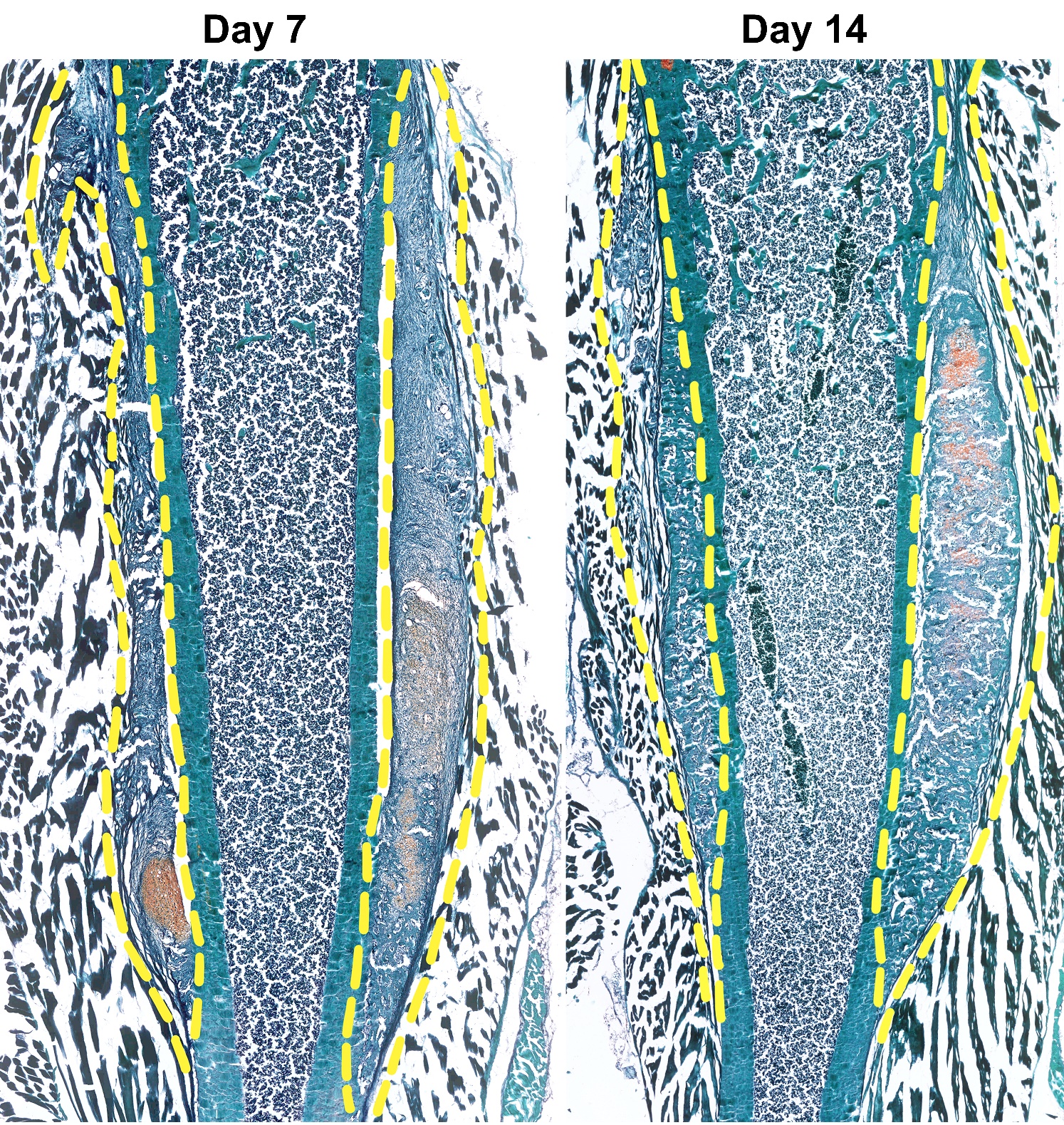
**

**Supplementary Figure 1.** **Full length femur histology following periosteal injury.**

Brightfield imaging of safranin O and fast green stained femur sections showing periosteal response following local injury at day 7 (mainly fibrocartilage formation and periosteum expansion) and day 14 (bone formation on the left side, cartilage and bone formation on the right side, both sides have marrow infiltration). Yellow dash line indicates the injured periosteum area and callus formation at the injury site.


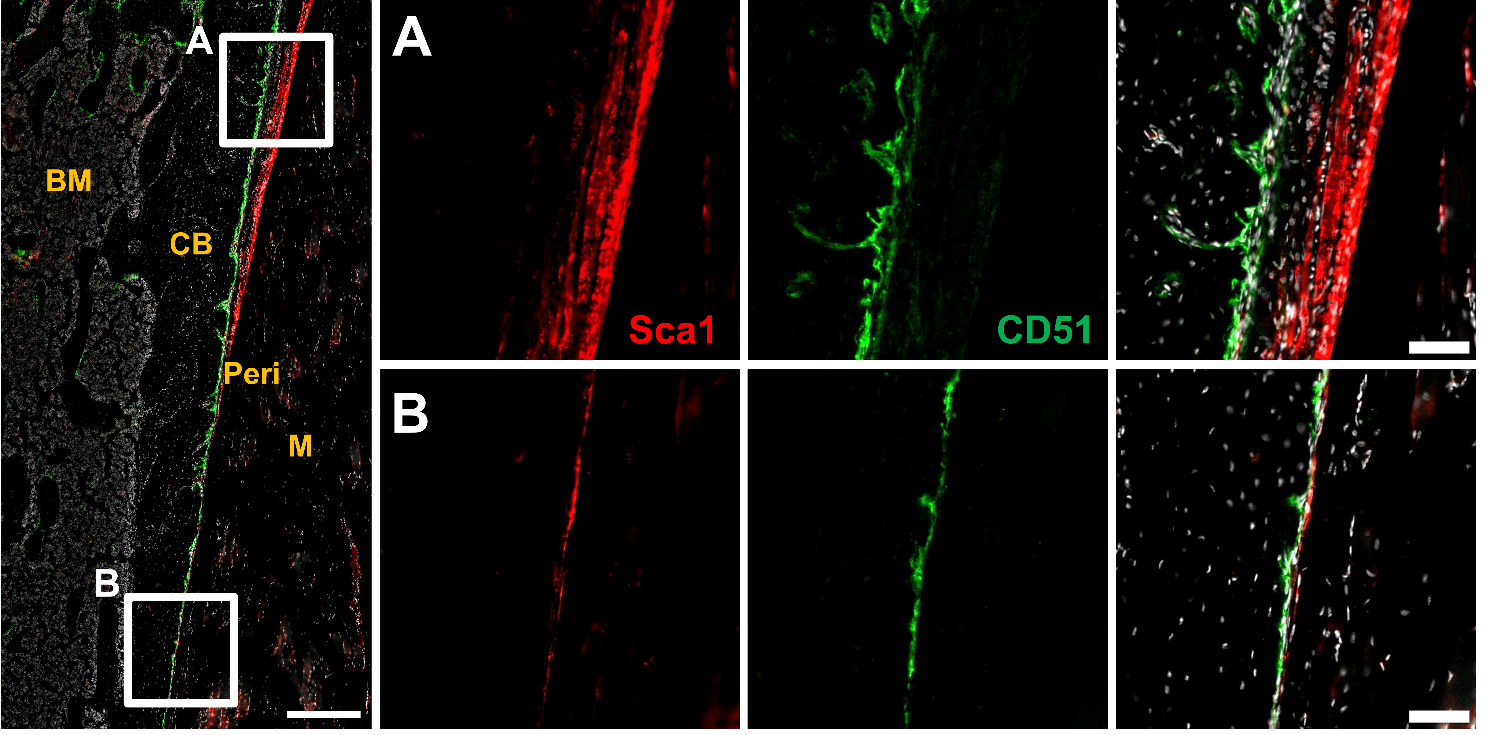
 Supplementary Figure 2. The localization of Sca1/CD51 populations in adult mice.

Representative histology showing the localization of Sca1/CD51 populations on the uninjured periosteum. DAPI (white), Sca1 (red), and CD51 (green) were labeled. Sca1 cells mainly resided in the outer layer of the periosteum, and CD51 cells localized in the inner layer of the periosteum. BM: bone marrow; CB: cortical bone; Peri: periosteum (injured periosteum and healing response); M: Muscle. Scale bars are 500 µm (large scale image) or 100 µm (A-B). DAPI, 4’,6-diamidino-2-phenylindole.


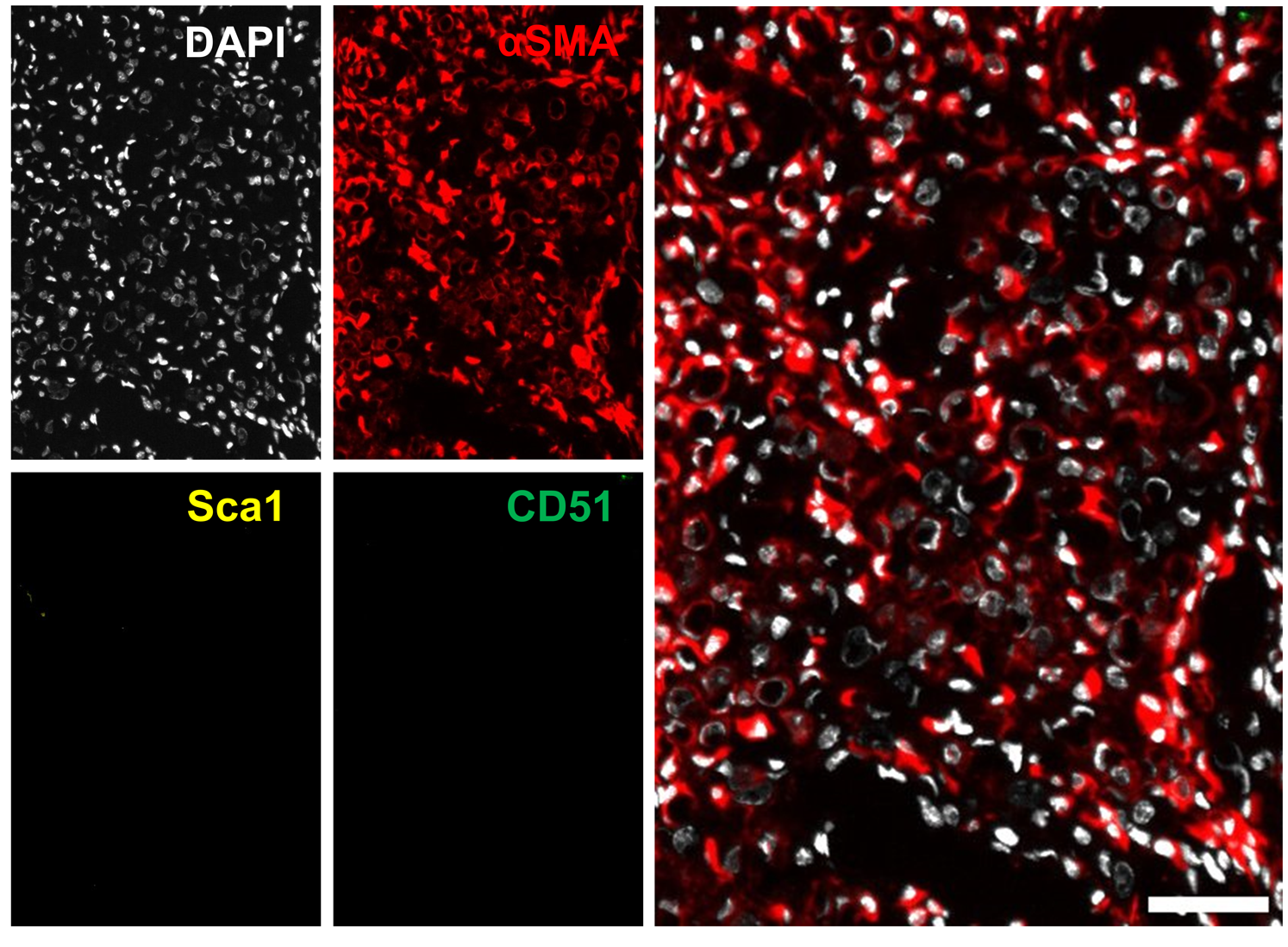


**Supplementary Figure 3.** **Minimal expression of stem and progenitor markers is found in injury-related fibrocartilage.**

Representative histology showing periosteum injury response at day 7 post scratch injury (n=4). DAPI (white), aSMA (red), Sca1 (yellow), CD51 (green). Chondrocytes did not express Sca1 or CD51. Scale bars are 50 µm. DAPI, 4’,6-diamidino-2-phenylindole.

## Supplementary Tables

**Supplementary Table 1. Antibodies, fluorophores, and dilutions used in multicolor spectral analysis panel.**

| **Antigen** | **Fluorophore** | **Clone** | **Cat#** | **Lot#** | **Manufacturer** | **Dose (μL/100µl)** |
| --- | --- | --- | --- | --- | --- | --- |
| - | GFP |  |  |  |  |  |
| CD200 | PerCP710 | OX90 | 46-5200 | 2142988 | eBioscience | 0.5 |
| - | tdTomato |  |  |  |  |  |
| CD24 | PE-Cy5 | M1/69 | 15-0242 | 2114978 | eBioscience | 0.1 |
| PDPN | PE-Cy7 | 8.1.1 | 127411 | B288412 | Biolegend | 1 |
| PDGFRα | APC | APA5 | 17-1401 | 2279166 | eBioscience | 1 |
| CD34 | eFluor™660 | RAM4 | 50-0341 | 2305277 | eBioscience | 2 |
| Sca1 | AlexaFluor700 | D7 | 108142 | B309397 | Biolegend | 0.5 |
| CD90 | APC-eFluor780 | 53-2.1 | 47-0902 | 2371181 | eBioscience | 0.5 |
| - | DAPI |  |  |  |  |  |
| CD45 | Pacific Blue | 30-F11 | 103126 | B314223 | Biolegend | 0.25 |
| CD31 | Pacific Blue | 390 | 102422 | B313033 | Biolegend | 0.25 |
| Ter119 | Pacific Blue | TER-119 | 116232 | B281640 | Biolegend | 0.25 |
| CD73 | BV421 | TY/11.8 | 127217 | B313188 | Biolegend | 1 |
| CD146 | BV605 | ME-9F1 | 740434 | 1113539 | BD | 1 |
| ALP | BV650 | B4-78 | 742712 | 1113542 | BD | 0.6 |
| CD51 | BV711 | RMV-7 | 740755 | 1113974 | BD | 1 |
| CD105 | BV786 | MJ7/18 | 564746 | 1095007 | BD | 1 |

**Supplementary Table 2. Additional antibodies for LSR II and Cell Sorting.**

| **Antigen** | **Conjugate** | **Clone** | **Cat#** | **Lot#** | **Manufacturer** | **Dose (μL/100µl)** |
| --- | --- | --- | --- | --- | --- | --- |
| CD51 | biotin | RMV-7 | 13-0512 | 2049444 | eBioscience | 1 |
| CD34 | eFluor660 | RAM4 | 50-0341 | 2305277 | eBioscience | 2 |
| CD90 | BV605 | 53-2.1 | 563008 | 1005942 | BD | 1 |
| CD45 | FITC | 30-F11 | 35-0451 | C0451031517353 | Tonbo | 0.5 |
| CD45 | FITC | 30-F11 | 11-0451 | 2306900 | eBioscience | 0.5 |
| CD31 | FITC | 390 | 11-0311 | 2086274 | eBioscience | 0.25 |
| CD31 | eFluor450 | 390 | 48-0311 | 4301770 | eBioscience | 0.5 |
| Ter119 | FITC | TER-119 | 35-5921 | C5921080316353 | Tonbo | 0.5 |
| Ter119 | FITC | TER-119 | 11-5921 | 2197090 | eBioscience | 0.5 |
| Ter119 | eFluor450 | TER-119 | 48-5921 | 1974934 | eBioscience | 0.25 |
| **Secondary antibody** | | |  |  |  |  |
| Streptavidin APC eFluor 780 | | | 47-4317 |  | eBioscience | 0.25 |

**Supplementary Table 3. Other reagents used in multicolor spectral analysis.**

| **Antigen** | **Cat#** | **Lot#** | **Manufacturer** |
| --- | --- | --- | --- |
| TruStain FcX™ PLUS (anti-mouse CD16/32) Antibody | 156603 | B324053 | Biolegend |
| True-Stain Monocyte Blocker™ | 426103 | B330487 | Biolegend |
| Brilliant buffer | 566385 | 1018568 | BD Biosciences |
